# Therapeutic potential of HUC-MSC-exos primed with IFN-γ against LPS-induced acute lung injury

**DOI:** 10.22038/IJBMS.2023.74372.16156

**Published:** 2024

**Authors:** Chun Wang, Chen Jiang, Yiran Yang, Cheng Xi, Yunxiang Yin, Haiying Wu, Chuanyun Qian

**Affiliations:** 1 Kunming Medical University, Kunming, China; 2 Department of Emergency Intensive Care Unit, Second Affiliated Hospital of Kunming Medical University, Kunming, Yunnan, 650000, China; 3 Department of Gastrointestinal Surgery, First Affiliated Hospital of Kunming Medical University, Kunming, Yunnan, 650000, China; 4 Department of Emergency, First Affiliated Hospital of Kunming Medical University, Kunming, Yunnan, 650000, China

**Keywords:** Acute lung injury, Exosomes, Inflammation, Interferon gamma, Mesenchymal stem cells

## Abstract

**Objective(s):**

Human umbilical cord mesenchymal stem cells (HUC-MSCs) are pluripotent stem cells with anti-inflammatory and immunomodulatory properties used in the treatment of acute lung injury (ALI). However, the treatment of ALI using exosomes derived from HUC-MSCs (HUC-MSC-exos) primed with interferon-gamma (IFN-γ-exos) has not been described. This study investigated the effects of IFN-γ-exos on ALI.

**Materials and Methods:**

IFN-γ primed and unprimed HUC-MSC-exos (IFN-γ-exos and CON-exos, respectively) were extracted, identified, and traced. A549 cells and mice subjected to lipopolysaccharide (LPS)-induced inflammation were treated with IFN-γ-exos or CON-exos. Viability; interleukin (IL)-1β, IL-6, tumor necrosis factor (TNF)-α, and reactive oxygen species (ROS) levels; NF-κB p65, and NLRP3 expression and histology and lung injury scores were measured in cell, supernatant or lung tissue.

**Results:**

Indoleamine 2,3-dioxygenase (IDO) mRNA expression was elevated in HUC-MSCs primed with 5 ng/mL IFN-γ (*P*<0.001), and IFN-γ-exos and CON-exos were successfully extracted. LPS-induced inflammation resulted in decreased cell viability in A549 cells, and increased IL-1β, IL-6, TNF-α and ROS levels and NF-κB p65 and NLRP3 expression in A549 cells and mice(*P*<0.05 to *P*<0.001). Treatment with IFN-γ-exos and CON-exos increased cell viability and decreased the concentrations of IL-1β, and ROS, expression of NF-κB p65 and NLRP3, and the lung injury score, and these effects were more obvious for IFN-γ-exos(*P*<0.05 to *P*<0.001).

**Conclusion:**

IFN-γ-exos reduced oxidative stress and inflammatory responses in LPS-induced A549 cells and mice. The result demonstrated the therapeutic potential of IFN-γ-exos in LPS-induced ALI.

## Introduction

Acute lung injury (ALI) is an acute respiratory dysfunction caused by various infectious or non-infectious factors, such as sepsis, aspiration, and trauma. The prevalence of acute respiratory distress syndrome attributable to serious ALI (1, 2) is as high as 10% (3) in intensive care units. ALI is a global public health problem (4) resulting in three major outbreaks of infection occurring globally in the 21st century, namely severe acute respiratory syndrome in 2002, Middle East respiratory syndrome in 2012, and coronavirus disease 2019 in 2019, which has a mortality rate of 40%-50% (5, 6). Although most ALI survivors largely recover normal or near-normal lung function, they experience neuromuscular impairment, adaptations, and cognitive dysfunction, severely affecting their long-term quality of life (3). Therefore, it is critical and fundamental to investigate the pathophysiological processes of ALI and identify promising new therapeutic strategies. 

Exosomes (exos) are extracellular vesicles ranging in diameter from 30 to 150 nm that contain bioactive compounds, such as mRNA, miRNA, circRNA, peptides, cytokines, lipids, and proteins (7), which are important transmitters of intercellular interactions involved in physiological processes and diseases (8). Exos derived from MSCs (MSC-exos) have demonstrated immunomodulatory and tissue-healing capabilities comparable to the origin of MSCs (9). A previous study (10) found that adipose-derived MSC-exos reduced lung inflammation by inhibiting interleukin (IL)-27 secretion from macrophages, and exerted a protective effect against lung injury in septic mice. Therefore, MSC-exos represent a cell-free therapy for inflammatory diseases (11, 12).

However, it is worth noting that the characteristics and functions of MSC-exos vary depending on their origin and microenvironments (13). Interferon-gamma (IFN-γ) is an inflammatory factor released primarily by T-lymphocytes, that can alter the proteomics and metabolomics of MSCs, manifested as increased cell viability, the production of intracellular anti-pathogenic proteins, and enhanced anti-inflammatory and anti-fibrotic capacity (14). Although various pro-inflammatory cytokines have been identified to alter the function of MSCs, the recommendations of the International Society for Cellular Therapy (15) state that IFN-γ significantly promotes the expression of immunomodulatory-related genes such as indoleamine 2,3-dioxygenase (IDO), programmed cell death-ligand 1, histocompatibility locus antigen, and antibacterial and anti-viral genes in MSCs (16). Consequently, IFN-γ is a potential modulator of the immune function of MSCs. 

Based on previous clinical research (5, 17), human umbilical cord mesenchymal stem cells (HUC-MSCs) reduce cellular inflammatory factors, alleviate the inflammatory response, and improve respiratory function and clinical symptoms. In addition, HUC-MSCs are currently the cells of choice in regenerative medicine because of their non-invasive isolation, lack of ethical controversy, minimal immunogenicity, rapid self-renewal, and proliferation (18). Worthy, the impact of interest is persistent because the patients were in the inflammatory microenvironment and the intravenous injection of HUC-MSCs was trapped in the capillary network (19). Thus, the actual mechanism of HUC-MSCs might differ, and their effects might be attributable to exos, particularly in the inflammatory microenvironment. Therefore, the novelty of our study is in the manipulation of cellular immunoregulatory actions by modifying the microenvironment of HUC-MSCs using IFN-γ, in addition to exploring whether functional exos released by HUC-MSCs are also altered and whether they exert a therapeutic effect on ALI.

## Materials and Methods

***Chemical reagents and cell experimental design***

HUC-MSCs were obtained from the Stem Cell Technology Application Research Center (Yunnan, China) and cultivated in a special serum-free medium (System Biosciences, Palo Alto, CA, USA) in a 5% CO_2_ incubator at 37 °C. IFN-γ was acquired from PeproTech Inc. (Cranbury, NJ, USA). HUC-MSCs were assigned to four groups and primed with 0, 5, 50, or 100 ng/ml IFN-γ for 24 hr. Exos were isolated from the supernatant of unprimed HUC-MSCs or HUC-MSCs that had been primed with 5 ng/ml IFN-γ, and these exos were designated as CON-exos and IFN-γ-exos, respectively, for follow-up experiments.

A549 cells (Procell Life Science & Technology, Wuhan, China) were maintained in Dulbecco’s modified Eagle’s medium (Gibco, Thermo Fisher Scientific, Waltham, MA, USA) supplemented with 10% fetal bovine serum and 1% 100 U/ml penicillinstreptomycin (Gibco) in a 5% CO_2_ incubator at 37 °C.

The ALI cell model was constructed *in vitro* by inducing A549 cells, which were seeded in a six-well plate at a density of 5 × 10^5^ cells/well (2 ml per well), with 0.01, 0.1, 1, or 10 µg/ml lipopolysaccharide (LPS, Sigma-Aldrich, St. Louis, MO, USA) for 24 hr. After constructing the ALI cell model, five cell groups were created: CON (normal control cells), LPS (cells were induced with 1 μg/ml LPS for 24 hr), LPS + PBS (LPS-induced cells were treated with an equal volume of phosphate-buffered saline [PBS] for 24 hr), LPS + CON-exos (LPS-induced cells were treated with CON-exos at 50 μg/ml for 24 hr), and LPS + IFN-γ-exos (LPS-induced cells were treated with IFN-γ-exos at 50 μg/ml for 24 hr).

***Reverse transcription-quantitative polymerase chain reaction (RT-qPCR)***

Total RNA was extracted from HUC-MSCs primed with 0, 5, 50, or 100 ng/ml IFN-γ for 24 hr using RNAiso Plus (Takara Biomedical Technology Co., Ltd., Beijing, China). Nucleic acid concentrations were then assayed. A PrimeScript ^TM^ RT reagent kit with gDNA Eraser was used to eliminate the interference of gDNA and reverse transcribe the RNA into cDNA using the A-tailing method. The primer sequences for IDO and GAPDH (Tsingke Biology Co., Ltd., Beijing, China) are presented in Table 1. TB Green Premix Ex Taq^TM^ II was used for amplification, and qPCR was conducted using the Quantstudio 6 FlexReal Time PCR System. The CT values of different samples were recorded, and quantitative analysis of IDO mRNA in HUC-MSCs was performed using the 2^–^^ΔΔCt^ method with GAPDH as the intrinsic control.

***Exo extraction***

Exos were extracted from the supernatant of 3rd–5th passage unprimed or IFN-γ–primed (5 ng/ml) HUC-MSCs by ultrafiltration combined with successive differential centrifugation, and the exos were named with CON-exos and IFN-γ-exos, respectively. The supernatant was collected and centrifuged twice at 2000 × g and 10000 × g, respectively, for 30 min at 4 °C. The supernatant was decontaminated using a 0.22-μm sterile filtration membrane, placed in an ultrafiltration tube (MilliporeSigma, Burlington, MA, USA), paired, and centrifuged at 10000 × g for 30 min at 4 °C, and then 110,000 × g for 80 min at 4 °C in ultra-high-speed centrifuge tubes (Thermo Fisher Scientific Inc.), The supernatant was then suspended in 15 ml of pre-cooled PBS and centrifuged again. Finally, the precipitate was added to 70 μl of PBS repeatedly, dispensed, and stored at –80 °C for follow-up investigations.

***Identification of IFN-γ-exos and CON-exos***

The morphology of IFN-γ-exos and CON-exos was assessed via transmission electron microscopy (Leica, Carl Zeiss, Jena, Germany), in addition to the exo particle size, which was calculated using nanoparticle tracking analysis (Nano FCM). The exo-specific surface marker proteins TSG101, CD63, CD81, and calnexin were explored in IFN-γ-exos, CON-exos, and HUC-MSCs using western blotting. The bicinchoninic acid (BCA) method (Beyotime Biotechnology, Shanghai, China) was used to determine the protein concentration in IFN-γ-exos and CON-exos, and statistical significance was analyzed.

***Immunofluorescence staining***

HUC-MSC-exos were fluorescently tagged using the PKH26 dye kit (UmiBio Science and Technology Group, Shanghai, China) according to the manufacturer’s instructions. After completely mixing the PKH26 working solution with IFN-γ-exos and CON-exos solutions for 10 min, PBS was added and mixed, and PKH26-labeled IFN-γ-exos and CON-exos were isolated by successive differential centrifugation. 

A549 cells in suspension were counted and seeded at a density of 5 × 10^4^ cells/well in a 12-well plate, and PKH26-labeled IFN-γ-exos and CON-exos were added for 2 hr. Cells were fixed in 4% paraformaldehyde (Biosharp Life Sciences, Anhui, China), followed by nuclear staining with the anti-quencher DAPI (Biosharp Life Sciences). PKH26-labeled IFN-γ-exos and CON-exos were observed by monitoring a red fluorescence signal under an Olympus dx51 fluorescence microscope (Tokyo, Japan) at ×40, ×200, ×400.

Mouse lung tissues from each group were obtained and dehydrated in sucrose solution with a 15%–30% gradient. Subsequently, they were OCT-embedded, sectioned, circled with an immunohistochemical pen, dripped with reactive oxygen species (ROS; Beyotime Biotechnology) staining solution for 3–5 min, washed in PBST, sealed, fixed, and observed under a fluorescence microscope (Tokyo, Japan).

***Calculation of cell viability***

A549 cells were seeded into a 96-well plate at a density of 5 × 10^3^ cells/well. The complete medium was replaced with LPS working solutions (0, 0.01, 0.1, 1.0, or 10 μg/ml) for 24 hr. When screening therapeutic concentrations of IFN-γ-exos and CON-exos, the complete medium was replaced with 1 ug/ml LPS working solution for 24 hr, and then 5, 50, or 100 ug/ml of IFN-γ-exos and CON-exos were co-incubated for 24 hr. The Cell Counting Kit-8 (CCK-8, Dojindo Laboratories, Shanghai, China) working solution was formulated according to the manufacturer’s instructions, added at 110 μl/well, and incubated at 37 °C for 1–4 hr. Enzyme markers were used to calculate the optical density (OD) at 450 nm. Cell viability was calculated using the following formula: cell viability (%) = (A–B)/(C–B) × 100% (A: OD of the wells with cells, CCK-8 working solution, and medium; B: OD of cell-free wells containing medium and CCK-8 working mixture; C: OD of wells with cells, CCK-8 working mixture, and no drug). Data were subjected to statistical analysis.

***Western blot***

For western blotting, primary antibodies against NF-κB p65, nuclear erythro-derived factor 2 (Nrf2), NLRP3, IL-1β, GAPDH, and β-actin (1:1000, Abcam, Cambridge, UK) were used, and horseradish peroxidase-conjugated IgG (1:10,000, Proteintech Group Inc., Wuhan, China) was used as the secondary antibody. Cells were collected, washed twice with PBS, lysed in the lysis mixture with RIPA supplemented with phenylmethanesulfonyl fluoride, protease, and phosphatase inhibitor (Thermo Fisher Scientific) for 30 min at 4 °C, and then completely lysed using an ultrasonic wall breaker. Mouse lung tissues were processed in the same manner. The protein content was calculated using the BCA method. Protein extracts were electrophoresed by Tris-HCl-SDS-PAGE and subsequently mounted onto a PVDF membrane. The PVDF membrane was sealed for 1 hr with skimmed milk powder. The membrane was incubated with the specified primary antibodies overnight at 4 °C, followed by exposure to secondary antibodies for 1 hr. Chemiluminescence solution permeated the membrane, and detection was performed using a chemiluminescent imager. ImageJ was used to evaluate the gray value of the strips.

***Flow cytometry***

An ROS Assay Kit (Beyotime Biotechnology) was used to calculate the amount of ROS in the cells in each group. The cells were digested with 0.25% trypsin, and washed twice with PBS. ROS staining of the cells was performed in accordance with the manufacturer’s instructions. Fluorescence was monitored using a flow cytometer (BD Biosciences, Franklin Lakes, NJ, USA). Subsequently, the data were analyzed using the FlowJo program (v10.6.2).

***Animal experimental design***

All approaches adhered to the National Institutes of Health “Guide for the Care and Use of Laboratory Animals” and approved by the Animal Protection and Use Committee of Kunming Medical University (Approval number: kmmu20230914). Male C57BL/6 mice aged 6–8 weeks and weighing 20 ± 2 g were maintained in a pathogen-free environment. A longitudinal incision was made from the lower teeth to the center of the neck, where the tissue under the skin was clearly separated, revealing the windpipe. LPS solution (2.5, 5, or 10 mg/kg LPS) was instilled into the trachea of mice for 6 hr to identify the optimal induction concentrations for the constructing the ALI mouse models. We injected 25, 50, or 100 µl of exos via the tail vein to determine the optimal exo concentration. 

The mice were randomly assigned to five groups containing six mice each: CON (normal control group), LPS (ALI mice were instilled with 2.5 mg/kg LPS for 6 hr), LPS + PBS (ALI mice were constructed and injected with 25 μl of PBS via the tail vein for 24 hr), LPS + CON-exos (ALI mice were constructed and injected with 25 μl of CON-exos via the tail vein for 24 hr), and LPS + IFN-γ-exos (ALI mice were constructed and injected with 25 μl of IFN-γ-exos via the tail vein for 24 hr). Pentobarbital sodium (40 mg/kg) was injected intraperitoneally into the mice to induce anesthesia, after which the lung tissues were removed for western blotting and hematoxylin and eosin (HE) staining.

***Live imaging of small animals***

IFN-γ-exos and CON-exos were labeled with DiR (UmiBio Science and Technology Group) working solution, which was prepared according to the manufacturer’s instructions. IFN-γ-exos and CON-exos solutions were incubated with the DiR working solution for 30 min before being added to 15 ml of PBS, as previously described.

Mice were randomly assigned to five groups (n = 3 each): CON (normal fed mice). CON-exos injection (mice were injected with 20 μl of DiR-labeled CON-exos solution via the tail vein for 24 hr), ALI and CON-exos injection (ALI mice were constructed and injected with 20 μl of DiR-labeled CON-exos solution via the tail vein for 24 hr), IFN-γ-exos injection (mice were injected with 20 μl of DiR-labeled IFN-γ-exos solution via the tail vein for 24 hr), ALI and IFN-γ-exos injection (ALI mice were constructed and injected with 20 μl of DiR-labeled IFN-γ-exos solution via the tail vein for 24 hr). Mice were anesthetized with pentobarbital sodium via intraperitoneal injection, and lung tissues were dissected from the thorax before imaging by flattening on the IVIS Lumina III Small Animal Live Imaging with Living Image software, and the red fluorescent signal was observed.

***Histology and lung injury score***

Mouse lung tissues were fixed in 4% paraformaldehyde (pH 7.4) in 0.1 M PBS (pH 7.4) for 24 hr. Lung cross-sections were cut into a thickness of 5 μm. For histological analysis, HE staining (Beyotime Biotechnology) was performed on ALI mouse lung slides according to the manufacturer’s instructions. The scoring system for lung injury in the Guide of American Thoracic Society Documents (20) was used to determine the pathological changes as follow: score = [(20 × A) + (14 × B) + (7 × C) + (7 × D) + ((2 × E)]/(number of fields × 100), where A denotes neutrophils in the interstitial space, B denotes hyaline membranes, C denotes neutrophils in the alveolar space, D denotes airspaces being overrun by protein-based detritus, and E denotes thickening of the alveolar septum. A light microscope was used to view pathological alterations in the lung tissues.

***Enzyme-linked immune sorbent assay (ELISA)***

A mouse ELISA kit (Invitrogen, Waltham, MA, USA) was used to assess the levels of tumor necrosis factor (TNF)-α, IL-1β, and IL-6 in the sera of mice, whereas a human ELISA kit was used to detect the concentrations of inflammatory factors in the supernatant of A549 cells. All procedures were performed in accordance with the manufacturers’ recommendations. The OD was measured at 450 nm.

***Statistical analysis***

SPSS 13 was used to analyze statistical differences. All data are presented as the mean ± standard deviation (SD). Data analysis was repeated at least three times. The Shapiro-Wilk test, also known as the QQ plot, was used to normalize the data distribution. The two-sample* t*-test was used to compare data from both groups. Three or more groups were compared using one-way analysis of variance. The significance level was set at *P*<0.05.

## Results

***Changes of HUC-MSCs primed with IFN-γ and identification of IFN-γ-exos and CON-exos***

**Figure 1:**
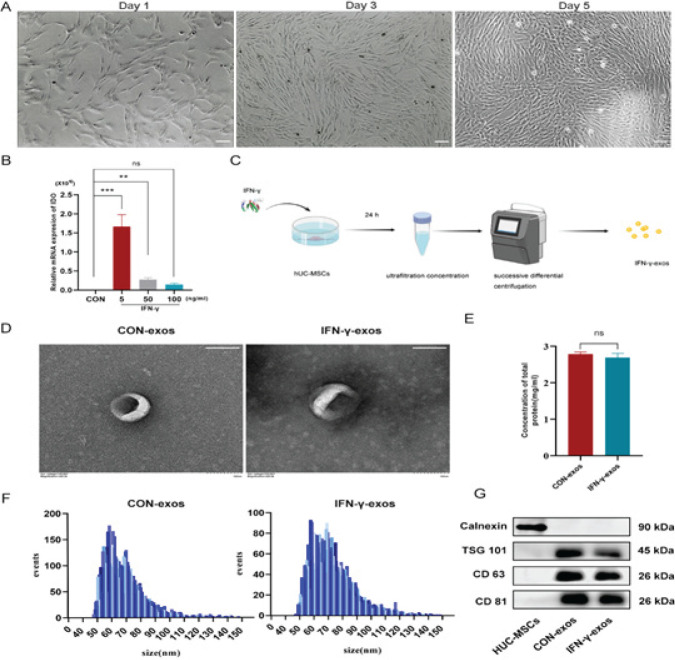
Changes of HUC-MSCs primed with IFN-γ and identification of IFN-γ-exos and CON-exos

HUC-MSCs were spindle-shaped with vigorous proliferation capabilities on days 1, 3, and 5 under normal culture conditions (Figure 1A). The RT-qPCR results demonstrated that at doses of 5, 50 ng/ml IFN-γ, considerably increased the expression of IDO mRNA in HUC-MSCs compared to that in the CON group, with the most significant elevation observed for 5 ng/ml IFN-γ (*P*<0.001, Figure 1B), indicating that HUC-MSCs were under immunological stress. Therefore, 5 ng/ml IFN-γ was chosen to target HUC-MSCs for the extraction of IFN-γ-exos in subsequent experiments. A schematic diagram of IFN-γ-exos and CON-exos extraction is presented in Figure 1C. The membranes of IFN-γ-exos and CON-exos were intact, and they had the typical “cup and disc” shape on transmission electron microscopy (Figure 1D). The average protein concentrations of IFN-γ-exos and CON-exos as determined by the BCA method were 2.69 and 2.79 μg/ml, respectively (Figure 1E). IFN-γ-exos and CON-exos ranged from 50 to 130 nm in diameter, and the particle concentration as measured by nanoparticle tracking analysis ranged 1×10^7^–1×10^11 ^particles/ml (Figures 1F). Both IFN-γ-exos and CON-exos commonly expressed CD81, CD63, and TSG101 but not calnexin, in contrast to HUC-MSCs, based on western blotting (Figures 1G). These experimental data confirmed that 5 ng/ml IFN-γ modifies the immunological function of HUC-MSCs and that IFN-γ-exos and CON-exos can be successfully extracted through ultrafiltration and successive differential centrifugation.

***Exo tracing in vitro and in vivo***

**Figure 2:**
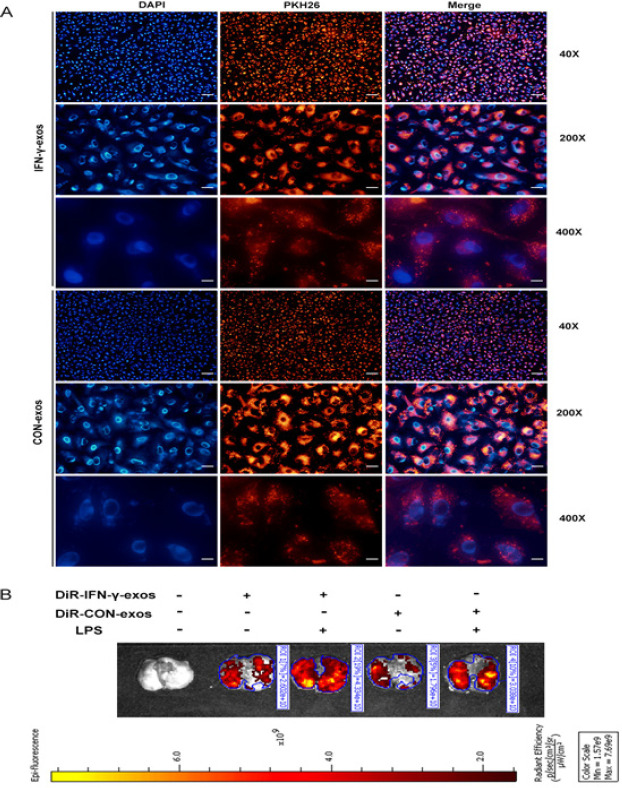
Exo tracing *in vitro* and *in vivo*

Fluorescence microscopy at ×40, ×200, and ×400 magnification was used to examine the fluorescence distribution of PKH26-labeled IFN-γ-exos and CON-exos in A549 cells, and red fluorescent signals denoting IFN-γ-exos and CON-exos were broadly disseminated in the cytoplasm (Figure 2A), indicating that IFN-γ-exos and CON-exos can be accumulated by A549 cells. The distribution of DiR-labeled IFN-γ-exos and CON-exos *in vivo* was investigated by small-animal live imaging, which revealed that red fluorescent signals denoting IFN-γ-exos and CON-exos were enriched in mouse lung tissues after tail vein injection, particularly in mice with ALI (Figure 2B). These results lay the groundwork for future research on the biological activities and processes of exos.

***Construction of the LPS-induced inflammatory damage model in A549 cells and screening for therapeutic concentrations of IFN-γ-exos and CON-exos in vitro***

**Figure 3:**
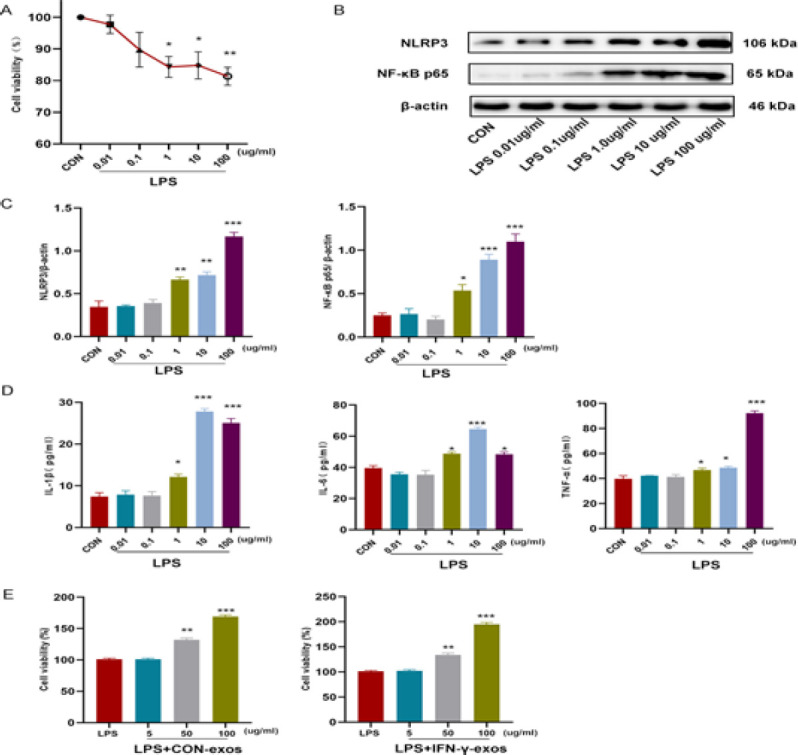
Construction of the LPS-induced inflammatory damage model in A549 cells and screening for therapeutic concentrations of IFN-γ-exos and CON-exos

The results demostrated that cell viability was decreased in the present of 1 μg/ml LPS (*P*<0.05, Figure 3A), and NF-κB p65 and NLRP3 expression was increased (*P*<0.05 to *P*<0.01). Meanwhile, IL-1β, IL-6, and TNF-α levles were all increased in the cell supernatants (*P*<0.05, Figures 3B–G). Consequently, 1 μg/ml LPS was used to construct an inflammatory damage cell model.

Cell viability was also used to screen the therapeutic concentration of exos in the LPS-induced cell inflammation model. The results indicated that in the presence of 50 μg/ml CON-exos and IFN-γ-exos, cell viability was significantly improved (*P*<0.01, Figure 3H, I). Therefore, 50 μg/ml was used as the therapeutic concentration in subsequent experiments.

***IFN-γ-exos attenuated inflammation and oxidative stress in LPS-induced ALI cells in vitro***

**Figure 4:**
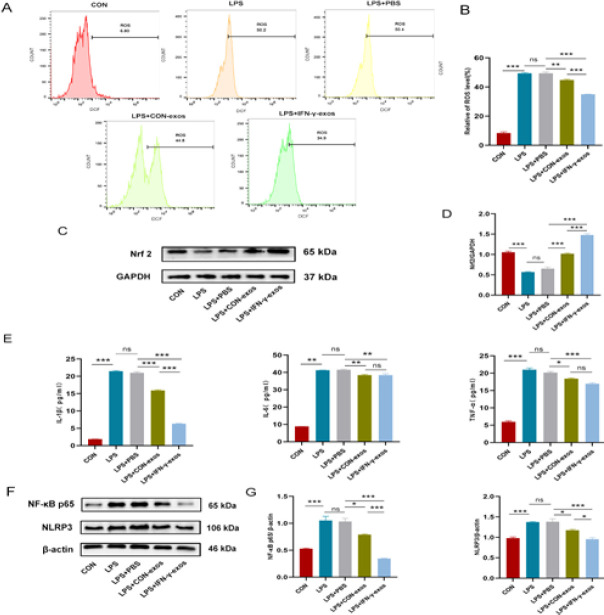
IFN-γ-exos attenuated inflammation and oxidative stress in the LPS-induced ALI cells model

The results indicated that both IFN-γ-exos and CON-exos significantly reduced ROS levels and Nrf2 protein expression (*P*<0.01 to *P*<0.001), with IFN-γ-exos having a more remarkable effect on oxidative stress (*P*<0.001, Figure 4A–D). 

According to ELISA data, IL-1β, IL-6, and TNF-α levels were considerably higher in the supernatant of ALI cell suspensions. Both IFN-γ-exos and CON-exos decreased the production of inflammatory factors in cells (*P*<0.05 to *P*<0.001). However, IFN-γ-exos had a significant inhibitory effect on IL-1β levels compared to the CON -exos (*P*<0.001, Figure 4E). In addition, the western blot results confirmed that IFN-γ-exos and CON-exos reduced the expression of NF-κB p65 and NLRP3 in the cells (*P*<0.05 to *P*<0.001), with IFN-γ-exos inducing a more obvious reduction (*P*< 0.001, Figure 4F, G).

***Construction of the LPS-induced ALI mouse model and screening for therapeutic concentrations of exos in vivo***

**Figure 5:**
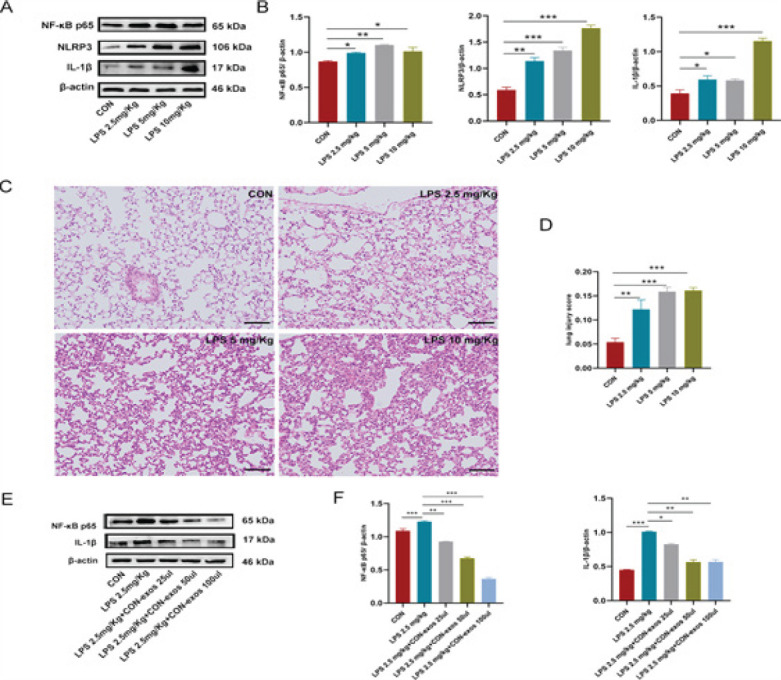
Construction of the LPS-induced ALI mouse model and screening for therapeutic concentrations of exos

In this study, an LPS concentration gradient was constructed to assess the impact of inflammation on mouse lung tissues using western blotting and pathological damage scoring. We discovered that at a dosage of 2.5 mg/kg LPS, the expression of NF-κB p65, NLRP3, and IL-1β was elevated in the lung tissues of mice compared to that in the CON group (*P*<0.05 to *P*<0.01, Figure 5A, B). The tissues severely disordered, with edema, hemorrhage, and alveolar collapse, and the pathological damage score was increased on HE staining (*P*<0.01, Figure 5C, D). The results indicated that 2.5 mg/kg LPS induced inflammatory damage in mouse lung tissues, and this concentration was used to construct the ALI mouse model.

To determine the therapeutic dose of exos, we administered several doses of CON-exos to ALI mice via the tail vein. The findings demonstrated that NF-κB p65 and IL-1β expression was elevated in the LPS group, implying that the ALI mouse model had been established (*P*<0.001), and their expression was decreased after treatment with 25 μl of CON-exos (*P*<0.05 to *P*<0.01, Figure 5E, F).Consequently, volume of 25 μl of CON-exos was selected in the follow-up experiments *in vivo*.

***IFN-γ-exos suppressed inflammation and oxidative stress in the ALI mouse model in vivo***

**Figure 6:**
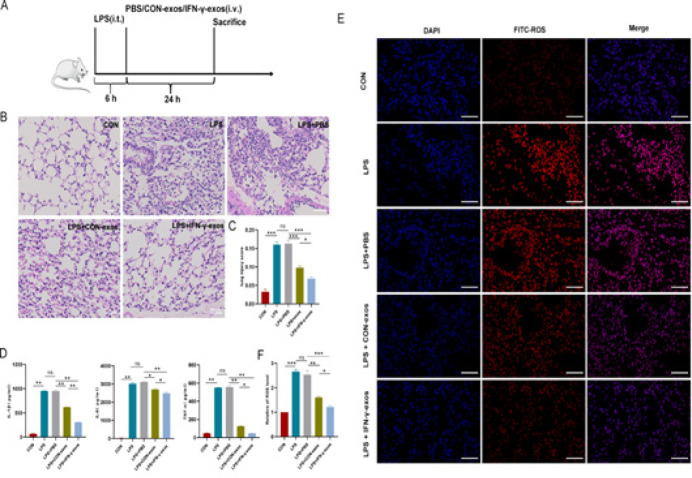
IFN-γ-exos suppressed inflammation and oxidative stress in the ALI mouse model

IFN-γ-exos and CON-exos, which were administered to ALI mice via the tail vein, reduced lung tissue exudation, hemorrhage, and structural disorders, as well as lung injury scores, with IFN-γ-exos exerting the strong effects (*P*< 0.001, Figure 6B, C). Moreover, the ROS level in mouse lung tissues was suppressed by IFN-γ-exos and CON-exos to the same extent as IL-1β, TNF-α, and IL-6 levels (*P*<0.05 to *P*<0.001), and the effect was more pronounced for IFN-γ-exos (*P*<0.01 to *P*<0.001, Figure 6D–F). These results are consistent with the* in vitro* data. 

## Discussion

HUC-MSCs have demonstrated immunological flexibility (21), as major histocompatibility complex-II and the co-stimulating molecules CD80 and CD86 were suppressed by immunomodulatory factors such as TGF-β and IDO after HUC-MSCs were primed with IFN-γ. For instance, the expression of immunomodulatory factors differs among species. Specifically, IDO is highly expressed in humans, whereas the opposite holds in mice (22). In this study, relatively modest IDO expression was observed in HUC-MSCs, and its expression dramatically increased after the cells were primed with IFN-γ. However, the effect was not concentration-dependent, implying that the immune function of HUC-MSCs is regulated by microenvironmental factors that might simultaneously regulate the composition of exos.

Exos are endonucleosome-derived organelles fused with the plasma membrane that are released extracellularly (23). The characteristics of exos including their fundamental attacking capacity, modest immunogenicity, substantial modification adaptability, and biological obstacle permeability make them promising drug carriers (24) that can target cells and exert biological actions (25). Most intravenously injected MSC-exos aggregate primarily in the liver, spleen, and lungs (26). It was also reported (27) that MSC-exos administered intravenously in AKI mice accumulate primarily in inflammation-damaged kidneys. In our study, we found that exos were well taken up by cells and distributed in the cytoplasm after co-incubation for only 2 hr, which was related to the enrichment of cholesterol, sphingomyelin, and ceramide in the exo membrane (28-30). Furthermore, the enrichment of DiR-labeled IFN-γ-exos and CON-exos in lung tissue was more pronounced with nearly 2-fold stronger red fluorescent signals after the construction of the ALI mouse model. Whether this was attributable to chemotaxis in response to inflammation is unknown, and this will be explored in our future studies. Overall, the successful uptake of large amounts of exos by cells and lung tissues provides the foundation for their biological effects.

LPS-induced inflammatory damage (31) in A549 cells might result in the dysregulation of redox homeostasis, leading to excess ROS levels (32) and the destruction of proteins, lipids, and nucleic acids (33-35). Nrf2 is a transcription factor that affects the activity of several oxidative enzymes (NOX, NOS, XO, and CYP) and plays an anti-oxidant role in ALI (36). Prior reported (37) that Nrf2 upregulation inhibits activation of the NF-κB pathway, mitigates the generation of pro-inflammatory cytokines and chemokines, and reduces inflammatory damage (38). Zheng *et al*. (39) explored the curative properties of HUC-MSC-exos in a rat model of LPS-induced ALI and found that HUC-MSC-exos mediated the downregulation of FZD6 expression by miR-22-3p, thereby alleviating inflammation and oxidative stress in lung tissues. In our study, we constructed a model of cellular inflammatory injury induced by LPS and discovered that Nrf2 expression was considerably decreased, indicating the presence of oxidative stress injury, in line with the previous report (40). Treatment with IFN-γ-exos, increased the intracellular expression of Nrf2, suggesting the existent of potent active substances involved in anti-oxidant stress pathway or inhibition of the inflammatory pathway.

The NF-κB, PTEN/PI3K/Akt, Wnt/β-catenin, and TGF-β/Smad pathways are all involved in the molecular process of ALI (41, 42). NF-κB signaling is a crucial inflammation-regulating pathway that modifies the expression of pro-inflammatory factors, and it is closely linked to the expression of NLRP3 (43). In this study, we induced ALI in mice via LPS airway perfusion to induce direct injury in alveolar epithelial cells and promote inflammatory reactions and sepsis. This strategy quickens the development of lung injury faster with fewer doses of LPS administered and more closely matches the guideline (20) requirement of the development of ALI in animals within 24 hr. The pathogenic processes are complicated (44). Our observations included considerable increases in NF-κB p65, NLRP3, and IL-1β expression in LPS-induced ALI, as treported previously (45), indicating that the NF-κB signaling pathway participates in the ALI inflammatory reaction. Meanwhile, IL-1β, IL-6, and TNF-α levels were reduced after treatment with IFN-γ-exos and CON-exos, in which the bioactive substance exerts its biological function of reducing the inflammatory response by entering the pulmonary circulation. The prominent anti-inflammatory and anti-oxidant properties of IFN-γ-exos appear to be the consequence of the potent regulatory competencies of HUC-MSCs after immune modification.

The strengths of the study include the construction of the ALI mouse model by LPS airway perfusion, and the innovative idea to immunomodulate HUC-MSCs by IFN-γ to explore the protective effect of their released functional exos against ALI and the differences between IFN-γ-exos and CON-exos in terms of size, shape, particle concentration, and protein concentration. However, whether IFN-γ–stimulated HUC-MSCs undergo changes in exo content and exert therapeutic effects through regulatory changes of the IDO gene is not completely clarified. This study revealed a correlation, but further in-depth study requires overexpression and knockdown of the IDO gene and observation of the changes in the exo content as well as the therapeutic effects on ALI. Second, we found that exos exert their main therapeutic effects mainly through bioactive substances, but the composition of these substances has not been clarified, necessitating further in-depth research.

**Table 1:** The primer sequences for IDO and GAPDH genes

**Name**	**Sense**
IDO	Forward: 5′-TGGCCAGCTTCGAGAAAGAG-3′ Reverse: 5′-GATAGCTGGGGGTTGCCTTT-3′
GAPDH	Forward: 5′-ACGGCAAGTTCAACGGCACAG-3′ Reverse:5′-CGACATACTCAGCACCAGCATCAC-3′

(A). Morphology of HUC-MSCs on days 1, 3 and 5 under normal culture conditions (scale bar = 100 μm). (B). Changes in IDO mRNA levels in HUC-MSCs treated with 5, 50, or 100 ng/ml IFN-γ. (C). Flow chart of CON-exos and IFN-γ-exos extraction. (D). The morphology of CON-exos and IFN-γ-exos was detected under transmission electron microscopy (scale bar = 100 nm). (E). Protein concentrations in CON-exos and IFN-γ-exos were measured by the bicinchoninic acid method. (F). The diameter distribution of CON-exos and IFN-γ-exos was examined by nanoparticle tracking analysis. (G). Protein expression of TSG 101, CD63, CD81, and calnexin in HUC-MSCs, CON-exos and IFN-γ-exos was examined by western blotting. Bar represents the mean ± SD (n = 3 per group). Differences between each value were analyzed by Student’s t-test and three groups were compared by one-way analysis of variance (ns: *P*>0.05,*: *P*< 0.05,**: *P*<0.01,***: *P*<0.001).

HUC-MSCs: Human umbilical cord mesenchymal stem cells

(A). Uptake of PHK26-labeled IFN-γ-exos and CON-exos by A549 cells observed by fluorescence microscopy at ×40 (scale bar = 100 μm), ×200 (scale bar = 20 μm), and ×400 (scale bar = 10 μm). (B). DiR-labeled IFN-γ-exos and CON-exos in the lung tissues of mice with ALI or control mice were detected by small-animal live imaging.

ALI: Acute lung injury

(A). CCK-8 was applied to evaluate viability in A549 cells induced with 0.01, 0.1, 1, 10, or 100 ug/ml LPS. (B). The protein expression of NLRP3 and NF-κB p65 in LPS-induced A549 cells was identified using western blotting. (C). Relative expression of NLRP3 and NF-κB p65. (D). Enzyme-linked immunosorbent assay was used to detect the levels of the inflammatory factors IL-1β, IL-6, and TNF-α in the supernatants in response to a concentration gradient of LPS. (E). Viability of inflammation-damaged cells in the presence of different concentrations of IFN-γ-exos and CON-exos as determined by the CCK-8 assay. Bar represents the mean ± SD (n = 3 per group). Differences between three or more groups were compared using one-way analysis of variance. (ns: *P*>0.05,*: *P*<0.05,**: *P*<0.01,***: *P*<0.001).

LPS: Lipopolysaccharide; CCK: Cell counting kit

(A). ROS levels in each experimental group (CON, LPS, LPS + PBS, LPS + CON-exos, and LPS + IFN-γ-exos) *in vitro *were determined by flow cytology. (B). Relative ROS levels. (C). The protein expression of Nrf2 was determined in each experimental group by western blotting. (D). Relative expression of Nrf2. (E). IL-1β, IL-6, and TNF-α contents were detected by ELISA in the cell supernatant of each experimental group. (F). Protein expression of NF-κB p65 and NLRP3 in each experimental group was determined by western blotting. (G). Relative expression of NF-κB p65 and NLRP3. Bar represents the mean ± SD (n = 3 per group). Differences between three or more groups were compared using one-way analysis of variance. (ns: *P*>0.05,*: *P*<0.05,**: *P*<0.01,***: *P*<0.001).

LPS: Lipopolysaccharide; ROS: Reactive oxygen species; PBS: Phosphate-buffered saline

(A). Protein expression of NF-κB p65, NLRP3, and IL-1β in the lung tissues of mice induced with 2.5, 5, or 10 mg/kg LPS was detected by western blotting. (B). Relative expression of NF-κB p65, IL-1β, and NLRP3. (C). Hematoxylin–eosin staining of lung tissues under the LPS gradient concentration (scale bar = 100 μm). (D). Injury scores of lung tissues in mice with LPS-induced ALI. (E). Protein expression of NF-κB p65, and IL-1β in the lung tissues of ALI mice after the administration of different concentrations of CON-exos was detected by western blotting. (F). Relative expression of NF-κB p65 and IL-1β. Bar represents the mean ± SD (n = 3 per group). Differences between three or more groups were compared using one-way analysis of variance. (ns: *P*> 0.05,*: *P*<0.05,**: *P*<0.01,***: *P*<0.001).

ALI: Acute lung injury

(A). Schematic diagram of experiments exploring the therapeutic effects of IFN-γ-exos and CON-exos on ALI mouse models. (B). HE staining of the lung tissues of mice administrated IFN-γ-exos and CON-exos in each experimental group (scale bar = 50 μm). (C). Injury scores of lung tissues in each experimental group. (D). The contents of IL-1β, IL-6, and TNF-α in the sera of mice in each experimental group were measured by ELISA. (E). ROS levels in the lung tissues of ALI mice were assessed by immunofluorescence analysis (scale bar = 50 μm). (F). Relative ROS levels; Bar represents the mean ± SD (n = 3–6 per group). Differences between three or more groups were compared using one-way analysis of variance. (ns: *P*>0.05,*: *P*<0.05,**: *P*<0.01,***: *P*<0.001).

ALI: Acute lung injury

## Conclusion

This study revealed that IFN-γ-exos alleviated oxidative stress and inflammatory responses and exerted a therapeutic effect against ALI, possibly by altering the levels of bioactive substances such as miRNAs, lncRNA, and proteins. Exploration of the specific mechanism of action and the composition of the active substance are future goals of our future research program. This finding provides evidence for the use of stem cell-free therapy and genetic engineering strategies in the treatment of ALI. 

## Authors’ Contributions

Q CY and W HY designed the experiments; W C, J C and Y YR performed experiments and collected data; X C and YYX discussed the results and strategy; Q CY and W HY supervised, directed, and managed the study; W C, J C, Y YR, X C, Y YX, Q CY and W HY finally approved the version to be published.

## Data Availability Statement

The raw data in this work are accessible upon reasonable request from the corresponding author.

## Funding

This work was supported by the National Natural Science Foundation of China (81960817 and 82260384) and the Kunming Science and Technology Bureau (2020-1-H-011).

## Ethics Approval

Animal experiments were performed in accordance with the Experimental Animal Care Guidelines and complied with the ethical criteria of the research ethics committee of the faculty of medicine at the Experimental Animal Center of Kunming Medical University, Kunming (Permission code: kmmu20230914).

## Conflicts of Interest

The authors declare that the research was conducted in the absence of any commercial or financial relationships that could be construed as a potential conflict of interest.
